# Cancer Immunoprevention and Public Health

**DOI:** 10.3389/fpubh.2017.00101

**Published:** 2017-05-08

**Authors:** Sandeep K. Singh, Mehmet Tevfik Dorak

**Affiliations:** ^1^Department of Biological Sciences, Florida International University, Miami, FL, USA; ^2^School of Health Sciences, Liverpool Hope University, Liverpool, UK

**Keywords:** cancer prevention, cancer immunology, cancer immune surveillance, immunoprevention, public health, cancer risk factors

## Abstract

The power of cancer immune surveillance has been documented beyond doubt, and the successful exploitation of immune response to cancer has started a new era in the war against cancer. Cancer biologists have recognized immunoevasion as an emerging hallmark in addition to the six hallmarks of cancer. Besides the natural connection between the immune system and cancer development, most established environmental risk factors are now known to interfere with immune surveillance mechanisms. Genetic variations regulating immunity may also modulate cancer susceptibility, but evidence for this is currently limited. Molecular cross talk linking “immune” and “genomic” surveillance pathways has been characterized. It appears that immune mechanisms may contribute to the effects of common cancer risk factors. We provide an updated overview of evidence for cancer immune surveillance, cancer risk factors interfering with it, and interventions to enhance cancer immune surveillance as tools to complement ongoing vaccine development efforts for cancer immunoprevention. Although there is a lot of support for cancer immunoprevention with simple lifestyle modifications from observational studies, there is an urgent need for clinical trials to establish the effectiveness of this approach for public health benefits.

## Introduction

Cancer is a major global public health issue. The global burden is continuing to increase and projected to reach 24 million in 2035 from 14 million new cases in 2012 (1). While major progress has been made in treatment, prevention remains to be the priority (1). Cancer prevention may benefit from the advances in cancer immunotherapy, which has led to the wider acceptance of the original idea of cancer immune surveillance. Indeed, the human body is not defenseless against cancer (2). The defense is provided by immune system and genomic surveillance mechanisms.

The original cancer immune surveillance concept was formulated almost half a century ago by Thomas (3) and Burnet (4, 5) [earlier studies are reviewed in Ref. (6–10)]. The cancer immune surveillance has now evolved into the cancer immunoediting concept (11), but for the exclusive consideration of cancer prevention, cancer immune surveillance is still a valid notion. The central theme of this idea was that an immune response can eliminate cells while they are still in the preclinical stages of transformation to overt cancer.

Given the prominent effect of immunotherapy in cancer treatment, and increasingly more widely accepted notion of cancer immune surveillance in cancer prevention (12–14), we explored potential connections between general cancer risk factors and immune capacity to examine whether the immune system may be a mediator for cancer risk. We also explored whether genetic epidemiology can be used as a probe for disease biology by checking genetic associations between immune system gene variant and cancer susceptibility. We review modifiable and non-modifiable lifestyle factors that influence the immune system and may be considered for cancer prevention.

## Immune System is Instrumental in Cancer Prevention

The relationship of the immune system to surveillance of cancer formation and treatment of clinical cancer has been extensively reviewed (11, 14–17). The evidence for cancer immune surveillance comes from animal studies, epidemiologic observations, and clinical observations.

### Animal Studies

The original immune surveillance hypothesis was experimentally tested in a nude mice model in the 1970s by Stutman at Memorial Sloan Kettering Cancer Center in New York. The expectation was that immunodeficient (athymic nude) mice should develop more spontaneous and carcinogen-induced tumors than their immunocompetent counterparts. Stutman’s experiments, however, did not yield results to validate these predictions (18, 19). Stutman’s study concluded, correctly, that their results argued against the thymus dependency of immunologic surveillance (19). The nude mice model was good as a T-cell-deficient model, but it was later recognized that they still had active natural killer (NK) cells and, therefore, were not completely immunodeficient (20). These results resulted in shelving of the cancer immune surveillance hypothesis for a while until a new series of experiments provided strong support in the 1990s. It is now well documented that the immune system has a critical role in controlling the development of not only virally induced tumors but also non-virally induced tumors (14, 21, 22).

In the modern experiments, when mice genetically engineered to be deficient for various components of the immune system were assessed for the development of carcinogen-induced tumors, it was observed that tumors arose more frequently and/or grew more rapidly in the immunodeficient mice relative to immunocompetent controls. In particular, deficiencies in the development or function of CD8+ cytotoxic T lymphocytes (CTLs), CD4+ Th1 helper T cells, or NK cells each led to demonstrable increases in tumor incidence; moreover, mice with combined immunodeficiencies in both T cells and NK cells were even more susceptible to cancer development. The results indicated that, at least in certain experimental models, both the innate and adaptive cellular arms of the immune system are able to contribute significantly to immune surveillance and thus tumor eradication (23–25).

In addition, cancer cells from immunodeficient mice are not capable of initiating tumors in other immunocompetent hosts, but cancer cells from immunocompetent mice can initiate transplanted tumors in immunocompetent or immunodeficient hosts (23, 24). Such behavior has been interpreted as follows: highly immunogenic cancer cell clones are eliminated in immunocompetent hosts by the process of immunoediting with weakly immunogenic variants continuing to grow. Those weakly immunogenic cells giving rise to the tumor can also colonize other hosts both immunodeficient and immunocompetent. In immunodeficient hosts, however, the immunogenic cancer cells are not selectively eliminated and can survive the non-existing immunoediting process. When cells from tumors not subjected to immunoediting are transplanted into other genetically identical hosts, the immunogenic cancer cells are rejected by the competent immune systems of the new hosts (26).

When the host is immunocompetent and the tumor is immunogenic, cancer immune surveillance works most efficiently. This combination results in an active immune system as evident in the tumor microenvironment and also as systemic antibody response against the tumor antigens. Systemic response and its positive correlation with clinical outcome have been observed in colorectal cancer (antibodies against carcinoembryonic antigen), pancreas cancer [antibodies against mucin 1 (MUC1)], anaplastic large cell lymphoma (antibodies against anaplastic lymphoma kinase), and lung cancer (antibodies against zinc-binding α2-glycoprotein-1) (27). The constitution of the immune cells in the tumor microenvironment also correlates with clinical outcome. The stronger the tumor-specific immune response, the better the outcome (14, 16, 21, 27–32).

The modern experiments resulted in that a whole series of phases in the interaction of the immune system and cancer make up the “immunoediting” process. These phases are elimination, equilibrium, and escape (11, 17, 33). Immunoediting process eliminates highly immunogenic tumors in their early phases and selects for less immunogenic ones allowing their escape from elimination. This happens both before (34) and after (35) treatment. It is the neoantigens derived from somatic mutations in tumors that provide the link between the adaptive immune system that mounts specific immune response and cancer elimination. The immunologic pressure against neoantigens is the reason for both immunological control (early) and escape from this control (late) in cancer development. Direct support for the immunoediting process also came from the observation that the more immunodeficient the host is, the more immunogenic their tumors are (17).

The immune system may also be a double-edged sword and act to induce or promote cancer *via* inflammation (36, 37). Either infection or autoimmunity-induced, or due to the lack of physical activity or obesity, chronic inflammation is a well-established risk factor for cancer development. Chronic inflammation is a feature of aging and increases the risk for cancer development, progression, and metastasis by generating a tumor-supporting microenvironment. Inflammation is recognized as a factor that fosters multiple cancer hallmark functions (38). Cancer’s abilities to thrive in a chronically inflamed microenvironment, evade immune recognition, and suppress immune reactivity are also named as three immune hallmarks of cancer (39).

### Epidemiologic Observations

Epidemiologic studies have provided strong evidence for the control of cancer formation and control by the immune system. The earliest observation was that people with a weakened immune system have higher cancer incidence rates. At least some of primary immunodeficiencies confer increased cancer risk (8, 40). Acquired immunodeficiencies or immunosuppression as happens in organ transplant recipients and HIV infection has increased risk for cancer (8, 41, 42). Although earlier studies only observed an increased risk for infection-related cancers, later and larger studies show an increased risk for non-infection-related cancers (42). Some immunosuppressed organ transplant recipients have been observed to develop donor-derived cancers, suggesting that in the ostensibly tumor-free donors, the cancer cells were held in check, in a dormant state, by a fully functional immune system (43). These observations provide examples for the elimination phase of the modern immunoediting process.

There is also evidence for the equilibrium phase. A convincing number of studies on people died of non-cancer causes have revealed a high degree of occult cancers in otherwise healthy individuals (44). Few specific examples are as follows: in 110 consecutive autopsies of women aged 20–54 years, 22 were found to have evidence of breast cancer (only 1 had a history of cancer). Of these, 45% had multifocal and 41% had bilateral evidence for cancer presence (45). Another study noted the presence of cancer in opposite breast in 80% of women who died with a clinical diagnosis of breast cancer (46).

Unexpectedly high *in situ* prostate cancer rates were also found in men. An evaluation of 152 prostate glands from young males aged 10–49 years, up to 44% of them (in the fifth decade of age) had microscopic evidence of prostate cancer with an increasing age-related incidence (47). In Hungary, incidental prostate cancer was found in 38.8% of 139 men aged 18–95 years, and there was also an age-related increase (48). A larger study examined 340 prostates harvested from organ donors and detected adenocarcinoma in 12% of them also with an age-dependent increase, leading to 1 in 3 chance of carrying incidental cancer in the 60- to 69-year-old age group and even higher (46%) in 70- to 81-year-old men (49). One study compared incidental prostate carcinoma rates between Russian (*n* = 220; mean age = 62.5 years) and Japanese (*n* = 100; mean age = 68.5 years) men in autopsy samples. Prostate cancer was detected in 37.3% of Russian and in 35.0% of Japanese men overall, with the cancer rates reaching more than 40% of men aged greater than 60 years and nearly 60% in men aged above 80 years (50). The prevalence of latent prostate cancer is well above 2.9% lifetime risk of dying from prostate cancer (51).

The autopsy findings in breast and prostate cancers suggest that our bodies harbor many more cancers than those become clinically evident [so-called cancer without disease (52)]. One way to interpret this observation is that most tumors are kept at equilibrium by the immune system. This interpretation also applies to the finding that pancreas cancer takes more than two decades to become metastatic and detectable (53). A similar phenomenon is reported for dormant melanoma in the mouse in which it was established that tumor cells disseminate early, but immunosurveillance limits metastatic outgrowth (54). There are, however, alternative interpretations involving angiogenesis pathways (52, 55). Thus, the reasons for the observations of dormant cancers at frequencies much higher than actual cancer incidence rates may include immune control, but this has not been specifically examined. Tumor dormancy as a result of endogenous immune surveillance is better documented for metastatic cells (54, 56), but there is currently no direct evidence for the role of the immune system in restriction of primary tumor growth in humans. This is, therefore, an area where future research may shed some light.

Clinical epidemiology also increasingly supports the existence of anticancer immune responses in some forms of human cancer (14, 16, 21, 27, 29–32). For example, patients who have cancer, especially colon and ovarian cancers, and who are heavily infiltrated with CTLs and NK cells have a better prognosis than those who lack such abundant killer lymphocytes.

Epidemiologic studies also suggested that otherwise healthy people with lower immune-mediated NK cell-mediated cytotoxic capacity develop more cancers in follow-up, and there is a genetic component in this correlation (57, 58). This is just additional evidence that NK cells play a critical role in the recognition and eradication of tumors (59). In experimental animal models, NK cell deficiency increases cancer occurrence just like observed in Chediak–Higashi syndrome characterized by abnormal NK cytotoxic function. Importantly, natural cytotoxicity correlates with lifestyle factors known to modify cancer susceptibility (60, 61). Among the statistically significant observations, smoking (negative effect on NK cell activity), green vegetable consumption, and regular sleeping (positive effect) (60) as well as personality types (61) were noteworthy. A separate study reported favorable effect of beta-carotene on NK cell function in the elderly (62).

### Clinical Observations

The strongest evidence for cancer immune surveillance comes from the incredible success of cancer immunotherapy, which has revolutionized cancer treatment. The Science magazine named cancer immunotherapy as the Breakthrough of the Year for 2013 (63). Besides cancer immunotherapy becoming a routine treatment choice, there are more than a dozen drugs or vaccines approved by the US Food and Drug Administration for cancer prevention (64). Cancer immunoediting, beginning with cancer immune surveillance and elimination and ending with escape, is now firmly established as a reality. As a result, escape from immune control (immunoevasion) is now one of the emerging hallmarks of cancer (38).

The success of immunotherapy comes from its ability to break down the immunoevasion caused by the tumor cells. Tumor cells interfere with the immune response against tumor antigens by releasing molecules, which are normally used by the immune system to self-limit the activation. Once the antigen receptors (T-cell receptors) are engaged with the antigen, in this case tumor antigen, T-cells also require an activation signal from the costimulatory molecule CD28 to become effective killer cells. It is the B7 molecule on the antigen-presenting cell that activates the costimulatory molecule CD28 on T cells. T cell activation is required for immune response but should be controlled to avoid overactivation and autoimmunity. Once activated, T cells start expressing coinhibitory immune checkpoint molecules, cytotoxic T lymphocyte-associated antigen 4 (CTLA-4), and programmed death 1 (PD-1) (65). Engagement of these molecules with B7 generates inhibitory signals for T cells. Tumor cells inhibit T cells and evade immune response against them by releasing such coinhibitory molecules. For the purpose of cancer immunotherapy, monoclonal antibodies have been generated to counter these inhibitory signals. This is achieved by “immune checkpoint blockade” of CTLA-4, PD-1, or PD-1 ligand (PD-L1). These are monoclonal antibodies known as ipilimumab (an inhibitor of CTLA-4), nivolumab and pembrolizumab (inhibitors of PD-1), and atezolizumab and durvalumab (inhibitors of PD-L1) (65). The success of these immune check point blockers is strong evidence for the involvement of the immune system in the control of cancer development.

Another clinical observation is that agents used in cancer prevention or treatment may stimulate the immune system. This is likened to “the invisible arm of immunity” acting alongside their cytotoxic effects. The chemoprevention agents such as repurposed drugs such as aspirin, COX-2 inhibitors (e.g., celecoxib), the antidiabetic agent metformin, the bisphosphonate zoledronic acid, and tamoxifen (and aromatase inhibitors) used in cancer prevention also show immune activity [reviewed in Ref. (32, 66)]. Another more experimental chemopreventive agent curcumin also has immunologic effects (32, 67). Likewise, the antitumor effects of many conventional cancer treatments also involve the immune system. They promote immunogenic tumor cell death or directly stimulate immunoeffector cells (27, 31, 32, 68). The immunologic effects include enhancing the antigen-presenting cell (mainly dendritic cells) activity and cross-presentation of tumor antigens to CD8+ T-cells (e.g., methotrexate), increasing HLA class I antigen expression (e.g., gemcitabine, oxaliplatin, cyclophosphamide), favorable modification of helper T-cell polarization, and inhibition of immuno suppressor cells [mainly FOXP3+ T regulatory (Treg) cells, and myeloid-derived suppressor cells (MDSCs)] (e.g., vincristine, docetaxel, paclitaxel) (16, 32). These observations even led to a new form of cancer treatment using low doses of conventional chemotherapeutic agents (metronomic chemotherapy) to stimulate the immune system as opposed to suppressing as they do at their usual doses (27). In fact, methotrexate is such a strong immunosuppressive so that it is also used to treat autoimmune disorders but is used as an immunostimulant at lower doses.

## Most Cancer Risk Factors Operate *via* the Immune System

### Environmental Risk Factors

#### Age and Sex

Age is one of the strongest risk factors for cancer development. It is reported that the mortality rate increases 43 times for cancer in people older than 65 years in Western countries (69). An examination of the US SEER Database (2009–2013) shows that the median age at diagnosis for all cancers is 65 years, and median age at death from cancer is 72 years (https://seer.cancer.gov/statfacts/html/all.html). Cancer occurs more frequently with increasing age due to cumulative exposures to carcinogenic agents such as tobacco, infectious agents, and chemicals, as well as due to physiologic changes in the body due to hormonal changes and immune system dysregulation. Among the physiologic changes, immunosenescence, which can be described as progressive decline of the immune functions, alongside qualitative and quantitative dysregulations involve both innate and adaptive arms of immunity (12, 70–74). Some of the consequences of immunosenescence with aging like reactivation of persistent viral infections (such as varicella zoster virus) are well recognized. Conversely, it is the persistent viral and parasitic infections that contribute to the loss of immunosurveillance *via* premature exhaustion of T cells (70). Overall, infections, cancer, and autoimmune diseases are more frequent in the elderly, and all are related to the changes in the immune system (71, 73). Most crucially, bone marrow hematopoietic stem cells skew toward myelopoiesis (and against lymphopoiesis) with age, which leads to increased numbers of MDSCs (73, 75). The increase in MDSC numbers show correlations with cancer incidence in the elderly (73, 75). Certain lifestyle factors may potentially contribute to age-associated unfavorably changes in immunity in the elderly, including psychosocial parameters, stress responsiveness, physical inactivity, and nutritional factors (76). Some of the age-related immune system changes in the elderly may be reversed by simple lifestyle modifications (76, 77), including aerobic exercise (78), which is further discussed below. Further, a lot of pharmacologic ways to rejuvenate the aging immune system are also present (72, 79), but none is in current routine use yet.

Sex is another consistent risk factor for cancer (80, 81). Sex effect is likely to operate through immune surveillance differences between the sexes (12, 82). Thus, like age, sex is another consistent risk factor that seems to operate *via* the immune system.

#### Lack of Physical Activity

Health benefits of regular and moderate intensity physical activity are not restricted to improvements in metabolic and cardiorespiratory function for prevention of type 2 diabetes and cardiovascular diseases. Physical activity also decreases the risk for various cancers by several mechanisms, including decreased sex hormones, metabolic hormones and inflammation, and quantitative and qualitative changes in immune cells (77, 83). Research on the effect of aerobic exercise on the immune response has confirmed the skeletal muscle as an endocrine organ capable of secreting cytokines, which are called “myokines.” Exercise modulates the host immune response *via* skeletal muscle–organ cross talk (84–86). Regular physical activity appears to have immunoenhancing effects *via* improved neutrophil microbicidal functions, which reduce the risk of infectious disease, and increased immune cell telomere length (77, 78, 87). Negative energy balance induced by exercise also exerts anti-inflammatory effects by reducing chronic low-grade inflammation (77, 88). Overall, the evidence for a causal association between physical activity and colon and breast cancers is strongest and somewhat weaker for prostate, lung, and endometrial cancers and insufficient for testicular and ovarian cancers (89, 90). As a result, more breast and colon cancers than coronary heart disease can be prevented by regular physical activity (90, 91). It has been estimated that up to 330,000 cases of 6 major cancers could have been prevented with regular physical activity at the recommended level in Europe in 2008 (90).

#### Obesity

Obesity is responsible up to 20% of cancer mortality (92). Both local and systemic mechanisms are involved in the mediation of increased cancer risk, and these include the immune system alterations associated with obesity (12). Like the muscle is now considered an organ, the adipose tissue is an active endocrine organ, and like myokines from the muscle, adipokines are released from the fat tissue, which target organs including the immune system (93, 94). Overall effects of obesity on the immune system can be summarized as direct negative effect of the major adipokine leptin on immune cells; induction of chronic systemic inflammation *via* secretion of pro-inflammatory adipokines; acceleration of the aging of thymus, an important immune system organ; adversely affect the function of the primary immune organs bone marrow and thymus *via* infiltration of fat tissue; and reduced immune response to infectious agents (12). In particular, the involution of the thymus with age and lower production of naive T cells contribute to lower immune surveillance in the elderly. It has been shown that obesity accelerates thymic involution resulting in lower T cell numbers (95). Therefore, obesity is another cancer risk factor that acts, among other mechanisms, through alterations it causes in the immune system.

#### Diet

The influence of dietary factors in the development of chronic diseases, including cancer, is well known. What is not that well known is the role of diet in predisposition to cancer *via* immune system modulation and anti-inflammatory effects (96). Candidate nutrients with immunomodulatory effects include essential fatty acids; antioxidants; calcium; zinc; selenium; vitamins A, D, B6, and folate; omega-3 polyunsaturated fatty acids (omega-3 PUFAs); glutamine; arginine; S-amino acids; nucleotides; polyphenols; epigallocatechin gallate; beta-glucans; isothiocyanates; curcumin; and probiotics, which make up immunonutrient mixes (96–98). Caloric restriction and protein calorie balance have also been reported to have some immunomodulatory effects. Unfortunately, despite some data from observational and animal studies, there are only limited data from randomized clinical trials (RCTs). A cohort study examined the effects of high marine omega-3 PUFAs intake on colon cancer occurrence and clinical status and reported a very favorable outcome with detectable improvement on the immune response to the tumor (99). A double-blind RCT explored the effectiveness of a commercially available immunomodulating enteral nutrition formula (Impact^®^, Nestlé) in radiochemotherapy-treated head and neck and esophageal cancer patients (100). By phenotyping, functional assays, and gene expression analysis, clear immunostimulation was noted although clinical correlates were not reported. Most interestingly, even cancer preventing effect of fiber may be due to its indirect effect on the immune system. High-fiber diet consisting of starch, cellulose, pectin, or fructan induce the gut microbiota to produce short-chain fatty acid metabolites (acetate, butyrate, propionate), which in turn stimulate Treg cells for expansion and immune-suppressive properties to control pro-inflammatory responses in the gut (101). Clinical correlates of this observation are yet unknown, but high-fiber diet may have an immunomodulatory component in its cancer preventive effects.

#### Smoking

Smoking and cancer connection is well established and is due to the carcinogenic content of tobacco products. The overall effects of smoking on the immune system is to weaken the immune system *via* a pro-inflammatory reaction and suppression of effector functions of immune cells as well as skewing of the immune response toward Th2 type (102) although the effect on cytokine production seems negligible (103). Cytotoxic T cells, NK cells, and macrophages are among important immune cells that are suppressed by smoking. It is probable that immune system alterations complement smoking-associated carcinogenic and mutagenic effects in the causal pathway to cancer development.

#### Childhood Infections and Future Cancer

Recurrent infections may show seemingly inconsistent correlations with future cancer risk. If recurrent infections are due to immunodeficiency, childhood cancer risk may be increased as a result of insufficient immune surveillance (104), but if they are community-acquired natural infections, these infections may help development of the immune system and subsequently prevent childhood cancer development (105). An abnormal immune response by an underdeveloped immune system to a common infection because of a delay in infectious exposure is also a possibility (106), which is known as the Greaves hypothesis proposed to explain the childhood leukemia occurrence most commonly in the age peak (2–5 years old).

The more interesting observations concern the lower risk of adult cancer following certain childhood infections (12). It has been observed that infections such as chicken pox, measles, pertussis, and mumps may lower the risk for a number of adult cancers, especially ovarian cancer (107, 108). This is attributed to molecular mimicry in which microorganisms causing these infections have certain antigens (such as MUC1) that induce a lifelong immunity, which then recognizes the same antigen on cancer cells. The preexisting immunity against tumor antigens due to cross-reactivity facilitates immune surveillance, and cancer would be prevented. An altered form of the tumor antigen MUC1 is frequently expressed in ovarian cancer, and immunity against MUC1 is formed by mumps infection, which results in the observed inverse correlation between mumps and ovarian cancer (107, 108). It is now a possibility that upward trends in cancer incidence may be contributed by the mass vaccination campaigns and fewer infectious episodes in childhood.

#### Reproductive Factors

Nulliparity is a strong and consistent risk factor for breast, ovarian, and endometrial cancers, whereas early full-term pregnancy and parity confer a significant protection. The mechanism is thought to involve local hormonal effects on these organs, but there appears to be an immune system involvement too (12). Comparative molecular studies in breast tissue have shown that the gene expression signature differences between nulliparous and multiparous women as well as between pregnancy and postpregnancy included those of immune surveillance genes (109, 110). It has also been proposed that pregnancy increases the exposure of women to fetal antigens and induces immunity against them, which may be targeted against cancers expressing fetal antigens (111). Equally, a greater number of ovulatory cycles increase the risk for ovarian, breast, endometrial, pancreas, and colon cancers and correlate with lower anti-MUC1 antibodies (107).

#### Other Environmental Cancer Risk Factors

Other connections of the immune system to cancer risk factors include pesticides (112), benzene (113), arsenic (114), ultraviolet irradiation (115) exposure, and psychological stress (116, 117).

### Genetic Risk Factors

Genetic variation is instrumental as the origin of variation observed in many traits. Formally, the contribution of heritable genetic variation to the phenotypic variance observed is measured by heritability, which lies between 0 and 100%. Heritability can be quantified in twin studies. Such a study of immune parameters concluded that heritability was less than 20%, and a great majority of examined parameters (>75%) were dominated by non-heritable, i.e., environmental, influences (118). Another twin study examined 78,000 immune traits of which 1,800 were independent and were subjected to a genome-wide search for genetic correlates (119). We have subjected known genetic markers of immune parameters (119, 120) to a cross examination against the existing genome-wide association studies (GWAS) result databases to see whether any of them is a cancer risk marker. As shown in Table 1, this effort only revealed very weak cancer associations in GWAS. When we repeated the same analysis with immunoregulatory gene SNPs reported to be associated with cancer risk in candidate gene studies, we observed stronger GWAS associations although not replicating the originally observed associations (Table 1). Thus, we could only obtain limited evidence for the involvement of immune system-related genetic polymorphisms in the modification of cancer risk. This result is not necessarily surprising given that GWAS cannot readily capture gene–gene or gene–environment interactions (121), which are probably more relevant to the immune system. Since most cancers result from the combined effects of environmental factors and inherited susceptibilities and that only a few cancers are “solely” of genetic origin. The importance of the environment in cancer causation has implications for studies of genetic risk in cancer that is not sufficiently appreciated (122).

**Table 1:** **Immune regulatory gene polymorphisms and cancer susceptibility in GWAS and GRASP catalogs**.

Genetic variants	Main non-cancer associations	Cancer associations	Reference [PubMed ID number (PMID)]^a^
Genetic variants regulating immune cell levels^b^	Autoimmune disorders (type 1 diabetes, rheumatoid arthritis)	Neuroblastoma (rs6547705; *P* = 2.0E−02)	21124317

Genetic variants correlated with immune traits^c^	Autoimmune disorders (ulcerative colitis); hemoglobin A2 level	Neuroblastoma (rs10917750; *P* = 5.8E−04—rs723177; *P* = 9.6E−04—rs4657090; *P* = 1.2E−03—rs1934908; *P* = 3.8E−03) Prostate cancer (rs12359272; *P* = 8.2E−03)	21124317 23668334

Immunoregulatory gene variants associated with cancer risk in candidate gene studies^d^	Autoimmune disorders (rheumatoid arthritis, Crohn disease)	Non-melanoma skin cancer (rs12203592; *P* = 7.2E−14)	23548203
		Melanoma (rs12203592; *P* = 2.5E−05)	20602913
		Neuroblastoma (rs12203592; *P* = 4.9E−05)	21124317
		Breast cancer (rs12203592; *P* = 2.7E−06)	20453838
		Hodgkin lymphoma (rs2395185; *P* = 4E−31)	22286212
		Lung cancer (rs2395185; *P* = 9.5E−10)	23143601
		Acute lymphoblastic leukemia (rs17007695; *P* = 9E−07)	19176441
		Childhood acute lymphoblastic leukemia (rs5742909, *P* = 8E−04)	20189245
		Breast cancer (rs2296135, *P* = 3.7E−03)	23468962
		Non-Hodgkin lymphoma (rs1041981; *P* = 1.9E−02)	21471979

*^a^For cancer associations shown (PMID)*.

*^b^Ref. (120) (SNPs listed in Supplementary Tables 5 and 6 in the original paper, *n* = 109. The SNP list is available in Table S1A in Supplementary Material in the present paper)*.

*^c^Ref. (119) (SNPs listed in Table 1/Supplementary Table 5 with *P* < 5E−10 in the original paper; *n* = 709. The SNP list is available in Table S1B in Supplementary Material in the present paper)*.

*^d^The SNPs included in the analysis here are listed in Table S1C in Supplementary Material*.

Cumulative evidence for immune surveillance against the development of cancer and for the control of existing cancer is listed in Table 2.

**Table 2:** **Evidence for immune surveillance against the development of cancer and for the control of existing cancer**.

Observation	Implication	Reference^a^
Immunodeficient animals and humans developing more cancers; immunosuppression causing an increase in cancer incidence	Control of cancer development by the immune system	(8, 40, 42, 123)
Increased risk for cancer in people with lower natural cytotoxic activity in their peripheral blood	Control of cancer development by the immune system	(57, 58)
A high percentage of occult cancers in autopsy studies	Control of cancer at the preclinical stage by the immune system (equilibrium)	(44–50)
Decades long time taken by pancreas cancer to become clinically overt	Control of cancer at the preclinical stage by the immune system	(53)
Control of occult cancer at the equilibrium phase by adaptive immune system	Control of cancer at the preclinical stage by the immune system	(124, 125)
Development of donor-derived malignancies that have been kept under control in immunosuppressed transplant recipients	Control of cancer at the preclinical stage by the immune system	(43)
More immunogenic tumors developing in more immunodeficient animals	Elimination of immunogenic tumors by immune competent hosts	(17, 124, 126)
Cancer patients with antibodies against antigens of their tumors (e.g., carcinoembryonic antigen, mucin 1) having a better clinical outcome and even spontaneous regression	Existent antitumor immunity exerting a favorable effect on the outcome	(27, 31, 127)
Successful chemopreventive agents (aspirin, metformin, tamoxifen, bisphosphonate) having immune enhancing effects	Contribution of the immune system to the preventive effects	(32, 66)
Successful cancer chemotherapeutic agents having immune modulatory effects favorable for anticancer immunity	Contribution of the immune system to the therapeutic effects	(27, 31, 32, 68)
The composition of tumor-infiltrating immune cells and indicators of immune system activity correlate with the clinical outcome	Existent antitumor immunity exerting a favorable effect on the outcome	(14, 16, 21, 27–32)

*^a^In some instances, review papers rather than primary references are cited*.

## Immunoprevention of Cancer as a Public Health Intervention

An immunologic approach to cancer prevention is not new (128). Most of the general cancer prevention approaches including elimination of environmental hazards (e.g., smoking), lifestyle changes (e.g., increased physical activity, healthy and balanced diet with green vegetables), chemopreventive agents in high-risk individuals, and obviously immunoprevention *via* vaccination [e.g., hepatitis B virus (HBV) and human papilloma virus (HPV) vaccination] target the immune system at least partially.

Specifically, immunoprevention aims to prevent cancers through the use of vaccines against carcinogenic viruses and tumor antigens, antibodies against immune molecules, and immune modulators (30). While vaccines against tumor antigens and antibodies are used in individuals with a specific cancer, immune modulators have more broad activities to enhance natural cancer immune surveillance. HBV and HPV vaccinations are approved interventions against liver and cervical cancer caused by these viruses, respectively. Pharmacologic immune modulators that are currently in use include aspirin, COX-2 inhibitors, aromatase inhibitors, metformin, and bisphosphonates (32). Non-pharmacologic effects linked to lifestyle factors and environmental exposures reviewed in this article may have a wider application in primary prevention in people who do not yet have cancer. In fact, now that cancer is becoming the most common cause of death in developed nations (129), and it is important to increase awareness about immunoenhancement and cancer immunoprevention for everyone’s benefit (78).

Immunoenhancement is a very active area of research (76–78, 87) with some emerging results (128). Among the non-pharmacologic interventions to enhance the immune system, physical activity, dietary factors, and lifestyle changes are the best-known ones, and the use of immune modulation for prevention rather than therapy is becoming a reality (128).

Regular moderate physical activity is the most effective prevention strategy for a number of chronic diseases. Despite being very affordable, it is probably the most underutilized intervention. While the impact of physical activity on cardiovascular system is well recognized and a lot of interventions are implemented to enhance cardiovascular health, its connection to the immune enhancement and cancer prevention is not known as much. This is despite that the impact is actually greater on cancer prevention although this is not exclusively due to immune-enhancing effects (90, 91). Despite calls for exercise clinical trials in cancer prevention research (130, 131), more progress has been recorded in clinical trials on the effect of exercise on clinical outcome and survivorship of cancers than their prevention (132, 133). Since the evidence is strongest for colon and breast cancers (89, 90), at least for these ones, the modulation of the immune system with an exercise program may be used to enhance prevention and treatment outcomes (86). As mentioned above, physical activity is also effective in reversing age-related deterioration in the immune system (78). Typically, as we age, physical activity levels decline. Among the reasons for this are the lack of motivation for physical activity and unawareness of the health benefits, while awareness of unhealthy lifestyle, perceived susceptibility to disease as well as motivation towards lifestyle changes are important mediators of participation in lifestyle interventions (134).

After physical activity, nutritional modifications have the strongest immunomodulatory effects that may be used as a public health intervention (78). Flavonoids (such as green tea) are well known for their cancer preventive properties, which are due to epigenetic modifications (135). Quercetin, for example, is proposed as a preventive dietary factor for melanoma (136), and green tea is an acknowledged cancer preventive agent in Japan (137). A positive effect of all flavonoids is modulation of the immune system (138). Thus, flavonoids, including green tea, can be considered as safe cancer immunopreventive agents for general use. Fiber- and green vegetable-rich diet is also safe for general use for their cancer preventive properties, which include immune enhancement. Dietary supplements mentioned above, including beta-carotene and vitamin E, are suggested as immunomodulatory agents, but confirmatory evidence from clinical trials is currently missing. Until then, the recommendation of the American Institute for Cancer Research of not to take any supplements for cancer prevention should be adhered to.

Although RCTs are regarded as the source of strongest evidence to support clinical decisions, increased trial complexity and cost may be inhibitory and may explain the scarcity of such trials in cancer immunoprevention studies. Recently popularized pragmatic clinical trials represent a new approach for enhancing the evidence base by relaxing the strict regulations governing RCT conduct (139, 140). These trials are more widely accessible by participants, are less resource intensive, and place minimal burden on participants. Pragmatic trials are designed to test the effectiveness of usually non-pharmacologic intervention in routine practice and whether an intervention actually works in real life. They, therefore, generate evidence with a greater external validity. Besides pragmatic trials, other more practical approaches such as systems science methods are also available as alternatives and have already been used to answer important public health science questions (141). Thus, implementation of cancer immunopreventive strategies as public health interventions may be expedited using research methods emerging as alternatives to RCTs.

While cancer immunotherapy has revolutionized cancer treatment, cancer immunoprevention using vaccines and chemopreventive agents for selected high-risk people is also moving into the clinics (1, 66). Recent technological developments herald a new era in cancer detection and management that will also necessitate public health interventions. Soon, there will be more people under active surveillance than ever before, and recommendations to these people may include lifestyle modifications to maintain a healthy immune system as well as personalized risk stratification based on the assessment of their immune system. Preventing cancer in otherwise healthy, asymptomatic, and unsuspecting people will need to be carefully considered. If lifestyle modifications to enhance immune surveillance to prevent cancer sounds like a far-fetched idea, one has to remember the recent success stories in cardiovascular disease prevention by the same approach (142). Cancer-related mortality is beginning to exceed cardiovascular disease-related mortality in some European countries (143) and US states (129), and primary prevention is the most effective way to fight cancer (144). With more than 50% of cancers are hypothetically preventable through lifestyle modifications (145), the aim should be to increase this proportion with the implementation of cancer immunoprevention strategies in the near future.

## Author Contributions

Substantial contributions to the conception or design of the work, drafting the work or revising it critically for important intellectual content, and agreement to be accountable for all aspects of the work in ensuring that questions related to the accuracy or integrity of any part of the work are appropriately investigated and resolved: MD, SS; the acquisition, analysis, or interpretation of data for the work: SS; and final approval of the version to be published: MD.

## Conflict of Interest Statement

The authors declare that the research was conducted in the absence of any commercial or financial relationships that could be construed as a potential conflict of interest.
